# Microbiota data from low biomass milk samples is markedly affected by laboratory and reagent contamination

**DOI:** 10.1371/journal.pone.0218257

**Published:** 2019-06-13

**Authors:** Josef Dahlberg, Li Sun, Karin Persson Waller, Karin Östensson, Mark McGuire, Sigrid Agenäs, Johan Dicksved

**Affiliations:** 1 Department of Animal Nutrition and Management, Swedish University of Agricultural Sciences, Uppsala, Sweden; 2 Department of Molecular Sciences, Swedish University of Agricultural Sciences, Uppsala, Sweden; 3 Department of Animal Health and Antimicrobial Strategies, National Veterinary Institute, Uppsala, Sweden; 4 Department of Clinical Sciences, Swedish University of Agricultural Sciences, Uppsala, Sweden; 5 Department of Animal and Veterinary Science, University of Idaho, Moscow, United States of America; University of Illinois, UNITED STATES

## Abstract

Discoveries of bacterial communities in environments that previously have been described as sterile have in recent years radically challenged the view of these environments. In this study we aimed to use 16S rRNA sequencing to describe the composition and temporal stability of the bacterial microbiota in bovine milk from healthy udder quarters, an environment previously believed to be sterile. Sequencing of the 16S rRNA gene is a technique commonly used to describe bacterial composition and diversity in various environments. With the increased use of 16S rRNA gene sequencing, awareness of methodological pitfalls such as biases and contamination has increased although not in equal amount. Evaluation of the composition and temporal stability of the microbiota in 288 milk samples was largely hampered by background contamination, despite careful and aseptic sample processing. Sequencing of no template control samples, positive control samples, with defined levels of bacteria, and 288 milk samples with various levels of bacterial growth, revealed that the data was influenced by contaminating taxa, primarily *Methylobacterium*. We observed an increasing impact of contamination with decreasing microbial biomass where the contaminating taxa became dominant in samples with less than 10^4^ bacterial cells per mL. By applying a contamination filtration on the sequence data, the amount of sequences was substantially reduced but only a minor impact on number of identified taxa and by culture known endogenous taxa was observed. This suggests that data filtration can be useful for identifying biologically relevant associations in milk microbiota data.

## Introduction

The introduction of DNA based methods to study bacterial communities has in recent years stimulated interest and substantially challenged previous knowledge about environments thought to be sterile. Milk, placenta and airways are examples of environments that previously were considered sterile in healthy individuals, but when studied with DNA based methods revealed to harbor their own microbiome [1–5]. Simultaneously publications on problems with laboratory and reagent contamination in microbiota studies have become increasingly common and a list of commonly occurring contaminating genera has been created [6, 7]. Occasionally discoveries of a microbiome in a previously believed sterile environments have been questioned and attributed to methodological artefacts [8].

Milk microbiota has been suggested to play an important role for infant gut development and maternal mammary gland health [9]. For the bovine mammary gland, a milk microbiota has been described [1] and associated to; somatic cell count (SCC) [10], culture negative mastitis samples [2], intra-mammary infection [11, 12],history of intra-mammary infection [13], farm environment [14] and cow genotype [15]. Recently the “logical implications” for a bovine milk microbiota has been questioned based on udder immunology and established models for mastitis control [16].

Sequencing of the 16S rRNA gene is the most commonly used technique to describe bacterial composition and diversity in various environments. 16S rRNA gene sequencing has revolutionized science but it is a challenging technique that is prone to introduction of errors and biases (see Pollock *et al*. [17] for review). Several published studies report occurrence of contamination in blank controls originating either from the reagents used to process samples or the laboratory environment [6–8, 18, 19]. Salter *et al*. [6] was among the first to suggest a correlation between microbial biomass and level of contamination. In their study, dilution series of a pure culture of *Salmonella bongori* became dominated with non-S*almonella* DNA after extraction and sequencing when input bacterial biomass was approximately 10^3^–10^4^ bacterial cells per mL. Glassing *et al*.[7] found similar results, in their study they extracted DNA from molecular grade water and determined DNA concentration using qPCR and universal primers. They reported contamination as 10 *Escherichia coli* equivalent genomes per μl in the absence of competing human DNA, corresponding to 10^4^ *E*. *coli* cells/mL. Subsequently, the “best practice” for microbiome studies based on sequencing of the 16S rRNA gene is constantly discussed [17].

In this paper we add information to the knowledge gap on how the microbiome profile in low biomass bovine milk samples is affected by sample processing.

The milk samples used in this study came from an animal experiment that was designed to assess the composition and temporal stability of the bovine milk microbiota in healthy udder quarters using 16S rRNA gene sequencing. Due to earlier reported technical challenges with samples containing a low bacterial biomass [6, 7] we sequenced the collected milk samples, negative controls, positive controls and used culturing data to evaluate; 1) how sample preparation and sequencing influence the bacterial composition, 2) the relation between cultivable bacteria and microbiota composition assessed by 16S rRNA gene sequencing and 3) level of contamination. Further we assessed two data filtration methods to exclude contaminating taxa from the data set.

## Material and method

### Animal study design

Nine cows in the dairy herd at the Swedish Livestock Research Center in Uppsala, Sweden were enrolled in the experiment. The cows were in lactation 1–3, day 187–316 in lactation at first sampling and had a milk SCC below 100 000 cells/mL in each udder quarter for six samplings during the three weeks prior to the start of the experiment. Milk SCC is used as a measurement of inflammation in the mammary gland and can also be used as an indicator of intramammary infection. SCC below 100 000 cells per mL is considered to indicate a healthy mammary gland. Quarter level milk samples were taken before morning milking on Mondays and Thursdays over four consecutive weeks. All cows were fed a standard diet with *ad libitum* silage and individual concentrate rations to meet the calculated nutrient requirements for their individual milk production. During the whole experiment all cows were kept in one group in a loose housing system, having access to the same type of bedding material, milked twice daily in an automatic rotary (DeLaval AMR, DeLaval AB, Tumba, Sweden) with 12 hour intervals. No antibiotics or other medication were given to the animals during the experiment or the three weeks preceding the experiment.

All animal handling was approved by the Uppsala animal ethics committee, protocol no: C99/13.

### Sampling and bacterial culturing

Milk samples were taken according to guidelines for bacteriological analyses [20]. Teats were wiped visually clean with an individual moist cloth, the teat apex was wiped with two alcohol soaked cotton wads, three squirts of milk were discarded before the milk sample was collected by hand milking into a sterile 15 mL tube and placed on ice. The collected milk samples were transported to a laboratory and gently mixed after reaching room temperature before being divided into five aliquots, each consisting of 2 mL of milk; four aliquots were frozen at minus 80°C and stored (for maximum 7 months) until sample preparation whereas the fifth aliquot was used for bacterial culturing and determination of SCC. The maximum time from sampling to freezing or bacterial culturing was 4.1 and 5.1 hours, respectively.

For bacterial culturing; 10 μl of milk was inoculated on agar plates with 5% bovine blood and 0.05% esculin (National Veterinary Institute, Uppsala, Sweden) and incubated aerobically at 37°C. Growth was evaluated after 24 and 48 hours as no growth 0–2 CFU/10 μl, sparse growth 3–10 CFU/10 μl, moderate growth 11–50 CFU/10 μl or abundant growth >50 CFU/10 μl. Plates with growth of >2 CFU were evaluated, and bacterial isolates were identified to species level using MALDI-TOF, when appropriate, at the ISO 17025 accredited Mastitis Laboratory at the National Veterinary Institute, Uppsala, Sweden. Milk SCC was measured on a DeLaval Cell counter (DCC DeLaval AB, Tumba, Sweden) with a fluorescent microscopy based method. Milk aliquots were processed and bacterial inoculation was performed on an ethanol cleaned bench top, only sterile equipment was used in contact with milk.

### DNA extraction

Milk aliquots were thawed, warmed to 20°C and vortexed at room temperature before 1 mL of milk was withdrawn for DNA extraction. The milk was centrifuged at 13 000 x g for 5 minutes, the supernatant and the fat layer was removed and DNA was extracted from the cell pellet using the PowerFood Microbial DNA isolation kit, kit batch no PF15C12, (MO BIO Laboratories, Inc., Carlsbad, USA) according to the manufacturer’s instructions except that a Mini-Beadbeater (Biospec products, Bartlesville, USA) was used for cell lysis. The bead beating step was performed 2 x 1 minutes at the setting homogenize. DNA extraction was performed in batches of 24 samples. For each DNA extraction batch an empty vial was used as a no-template DNA extraction control (NTC) into which the first reagent was added and further processed as the milk samples, i.e. one NTC per 23 extracted milk samples.

### 16S rRNA gene amplicon sequencing

Illumina MiSeq sequencing libraries were prepared by amplifying the V3–V4 region of the 16S rRNA gene using the 341F-805R primers described by Hugerth *et al*. [21]. The primers contained a linker sequence compatible with barcoding primers that were used to attach sample specific barcodes and Illumina adaptors in a second PCR. Each PCR reaction contained 12.5 μl of Phusion high-fidelity PCR master mix with HF buffer (Life technologies; Carlsbad, USA), 1.25 μl of each primer in a 10 μM solution, 5 μl DNA free water and 5 μl of DNA template. Thermocycling was performed on a MyCycler (Bio-Rad Laboratories Inc., Hercules, USA) and thermocycling conditions were: initial denaturation at 98°C for 30 sec, 35 cycles of denaturation at 98°C for 10 sec, annealing at 60°C for 30 sec and elongation at 72°C for 7 sec, a final elongation was performed at 72°C for 2 min after the last cycle. A positive and a negative PCR control were included in each run and the PCR reaction was repeated if the negative PCR control contained a band when visualised on 1% agarose gel. PCR products (20 μl) were purified with Ampure Beads (Beckman Coulter, Brea, USA) using 0.8 volumes of beads per volume of PCR product and eluted in (40 μl of) DNA free water. The second PCR attached Illumina adapters and barcodes; used the same thermocycling conditions for 10 cycles, 10 μl of purified PCR products as DNA template and one barcode per milk sample. PCR products were again purified with Ampure Beads but eluted in Elution Buffer. DNA was quantified with Qubit 3.0 Fluorometer (Life Technologies, Carlsbad, USA). The samples were thereafter pooled into equimolar amounts and sequenced on an Illumina MiSeq sequencer with v3 sequencing chemistry (Illumina Inc., San Diego, USA) at the Science for Life Laboratory (Uppsala, Sweden). The NTC’s from DNA extraction were included in all the steps of the 16S gene amplification. In the second PCR, all NTC reactions were run separately but a limited number of barcodes were used, i.e. the same barcode was used for several NTC. DNA extraction and first PCR preparations were performed in a laminar air-flow hood cleaned with 10% bleach and 70% ethanol, and UV-irradiated for 30 minutes before execution of sample processing.

### Mock community as positive control

Five commonly occurring udder pathogens were chosen to create a bacterial mock community used for method evaluation. *Escherichia coli* ATCC 25922, *Klebsiella pneumoniae* ATCC 13883, *Streptococcus dysgalactiae* CCUG 39323, *Staphylococcus aureus* ATCC 25923 and *Trueperella pyogenes* CCUG 39326 were cultured separately in 50 mL nutrient broth with 10% horse serum aerobically on a shaker at 37°C. The time of culture was 25 hours for *T*. *pyogenes* and 4 hours for the other bacteria. Bacterial concentrations were determined by manual counting of several aliquots from different dilutions using a Bürker counting chamber and a microscope with 100X enlargement. The five bacterial strains were used to create a mock community with equal numbers of cells and the mock community was prepared in three different dilutions (10^7^, 10^5^ and 10^3^ cells of each bacterial species per mL). DNA from the mock communities was extracted and 16S gene amplification was performed as for the milk samples except that a Precellys24 (Bertin Technologies, Montigny-le-Bretonneux, France, with cell disruption for 2 x 45 sec at 6500 rpm) was used for cell lysis during DNA extraction. Information on number of 16S rRNA gene copies per bacterial strain was obtained from the Ribosomal RNA Operon Copy Number Database (RRNDB) and NCBI GeneBank for accurate calculation of relative abundance of 16S rRNA genes in input data.

### Illumina sequencing data analysis

The generated sequencing data was processed according to the procedure described by Müller *et al*. [22]. Cutadapt tool [23] and Quantitative Insights into Microbial Ecology (QIIME) version 1.8.0 [24] was used to generate operational taxonomic units (OTUs) using the open reference OTU picking strategy at a threshold of 97%, with U-CLUST against a Greengenes core set (gg_13_8) [25, 26]. The representative sequences were aligned against the Greengenes core set using PyNAST software [27]. The chimeric sequences were removed by ChimeraSlayer [28]. Taxonomy was assigned to each OTU using the Ribosomal Database Project (RDP) classifier with a minimum confidence threshold of 80% [29]. The OTU table was further filtered to include OTUs present in at least three samples and randomly subsampled to contain 1498 reads per sample. After analysis of sequence data, genera that represented >1% of the total relative abundance in a NTC were identified as contaminants. Taxonomic families containing a contaminant were manually filtered out from the OTU table. A weighted UniFrac dissimilarity matrix was created in QIIME for the original and the filtered data. The UniFrac distances between samples were used to compare consecutively collected samples in bacteriologically stable quarters with randomly selected samples (i.e. samples taken from quarters with the same bacteriological finding by culture three or four days apart were compared to random values in the data set). This procedure was repeated both in the original and the filtered data set. In addition, the contamination identified herein were compared to the contamination identified by the “decontam” package in R [30].

Descriptive analysis on sequencing results and statistical analyses, multivariate analyses and contamination identification were performed using Microsoft Excel, PAST [31] and R [32] and statistical significance was set at the level P<0.05. The 16S rRNA gene sequences were deposited in the NCBI Sequence Read Archive (SRA) under accession number PRJNA485047.

## Results

### Udder health and bacterial growth in milk

**Fig 1:**
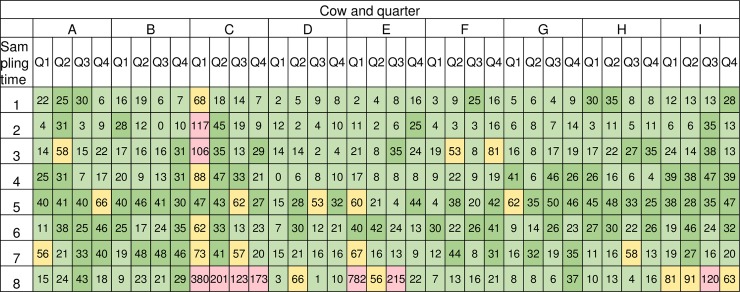
Milk somatic cell count (SCC) per cow, quarter and sampling time. Milk SCC expressed as x 1000/mL, cow (A-I) and quarter (Q1-Q4) in columns and sampling time (1–8) in rows. Light green; 0–24, dark green; 25–50, yellow; 51–100, pink; >100 cells/mL.

In this study the milk SCC of a majority of the quarters were stable and 96.9% (279/288) of the samples had a value below 100 000 cells/mL, averaging 17 195 cells/mL (Fig 1). The majority of the milk samples, 79.2% (228/288 samples), had no bacterial growth after 48 hours, 20 samples (6.9%) had sparse growth, 34 samples (11.8%) had moderate growth and six samples (2.1%) had abundant growth of bacteria after 48 hours (Table 1). Bacterial species identified by the Mastitis Laboratory at the National Veterinary Institute, Uppsala, Sweden were: *Corynebacterium* spp. (44 samples), mixed flora (15 samples) and *Staphylococcus* spp. (1 sample) (Table 1). Mixed flora was defined as growth of more than one phenotypically different CFU on the agar plate and were not further evaluated. Sixteen out of 36 quarters were bacteriological stable by culture throughout the study period, i.e. had the same bacterial species identified, or absence of bacteria, at all sampling points.

**Table 1:** Bacterial growth in 10 μl of milk per cow, quarter and sampling time.

	Cow and quarter																																			
	A				B				C				D				E				F				G				H				I			
Sampling time	Q1	Q2	Q3	Q4	Q1	Q2	Q3	Q4	Q1	Q2	Q3	Q4	Q1	Q2	Q3	Q4	Q1	Q2	Q3	Q4	Q1	Q2	Q3	Q4	Q1	Q2	Q3	Q4	Q1	Q2	Q3	Q4	Q1	Q2	Q3	Q4
1	0	C^2^	0	0	0	0	0	0	C^1^	C^2^	0	0	0	0	0	0	0	0	0	0	0	0	0	0	0	0	0	0	0	0	C^1^	0	0	0	0	0
2	0	0	0	0	0	0	0	C^1^	C^2^	C^1^	0	0	M^2^	0	0	0	S^1^	0	0	0	0	0	0	0	0	0	0	0	0	0	C^1^	0	M^1^	M^1^	0	0
3	0	C^1^	0	C^1^	0	0	0	C^1^	C^1^	C^1^	0	0	0	0	0	M^1^	0	0	0	0	C^1^	0	0	0	0	0	0	0	0	0	C^1^	M^1^	0	0	0	0
4	0	C^1^	0	0	0	0	0	0	C^2^	C^1^	0	0	0	M^1^	0	0	0	0	0	0	0	0	0	0	0	0	0	0	0	0	C^1^	0	0	0	0	M^2^
5	C^1^	C^1^	0	C^2^	0	0	0	C^1^	C^1^	C^1^	0	0	0	0	0	0	0	0	0	0	0	0	0	0	0	0	0	0	0	0	C^1^	0	0	0	0	0
6	C^1^	C^2^	C^1^	C^2^	0	0	0	0	C^2^	C^2^	0	0	0	0	0	0	0	0	0	0	0	0	0	0	0	0	0	0	0	0	C^1^	0	0	0	0	0
7	0	C^1^	C^2^	C^1^	M^1^	0	M^1^	C^1^	C^2^	C^1^	0	0	0	0	0	0	0	0	0	0	0	0	0	0	0	0	0	0	0	0	M^1^	M^1^	0	0	0	0
8	0	0	C^1^	C^3^	0	0	0	0	C^1^	C^1^	0	0	0	0	M^1^	0	0	0	0	0	0	0	0	0	0	0	M^1^	0	M^1^	0	C^2^	0	0	C^1^	0	0

Cow (A-I) and quarter (Q1-Q4) in columns and sampling time (1–8) in rows. 0 indicates no growth, C = growth of *Corynebacterium* spp, M = growth of mixed bacterial flora, S = growth of *Staphylococcus* spp. Superscripts ^1, 2, 3^ indicate sparse (3–10 CFU), moderate (11–50 CFU) and abundant (>50 CFU) growth, respectively.

### Microbiota in milk samples and negative controls

The 288 milk samples generated on average of 7 726 ±8355 quality controlled reads per sample (reads and DNA concentrations are provided in S1 Table). With the subsample threshold set to 1498 reads/sample, 278 milk samples were used for further analysis.

According to the sequencing results, four genera were present in more than 95% of all the milk samples; *Methylobacterium*, *Achromobacter*, *Burkholderia* and an unclassified genus in the family Oxalobacteriaceae. Together these genera represented 66% of the sequence data. *Methylobacterium* was the only genus present in all milk samples with average abundance of 57.9% (range 0.4–92.9%). Box plots for the ten most abundant genera are provided in S1 Fig.

**Fig 2:**
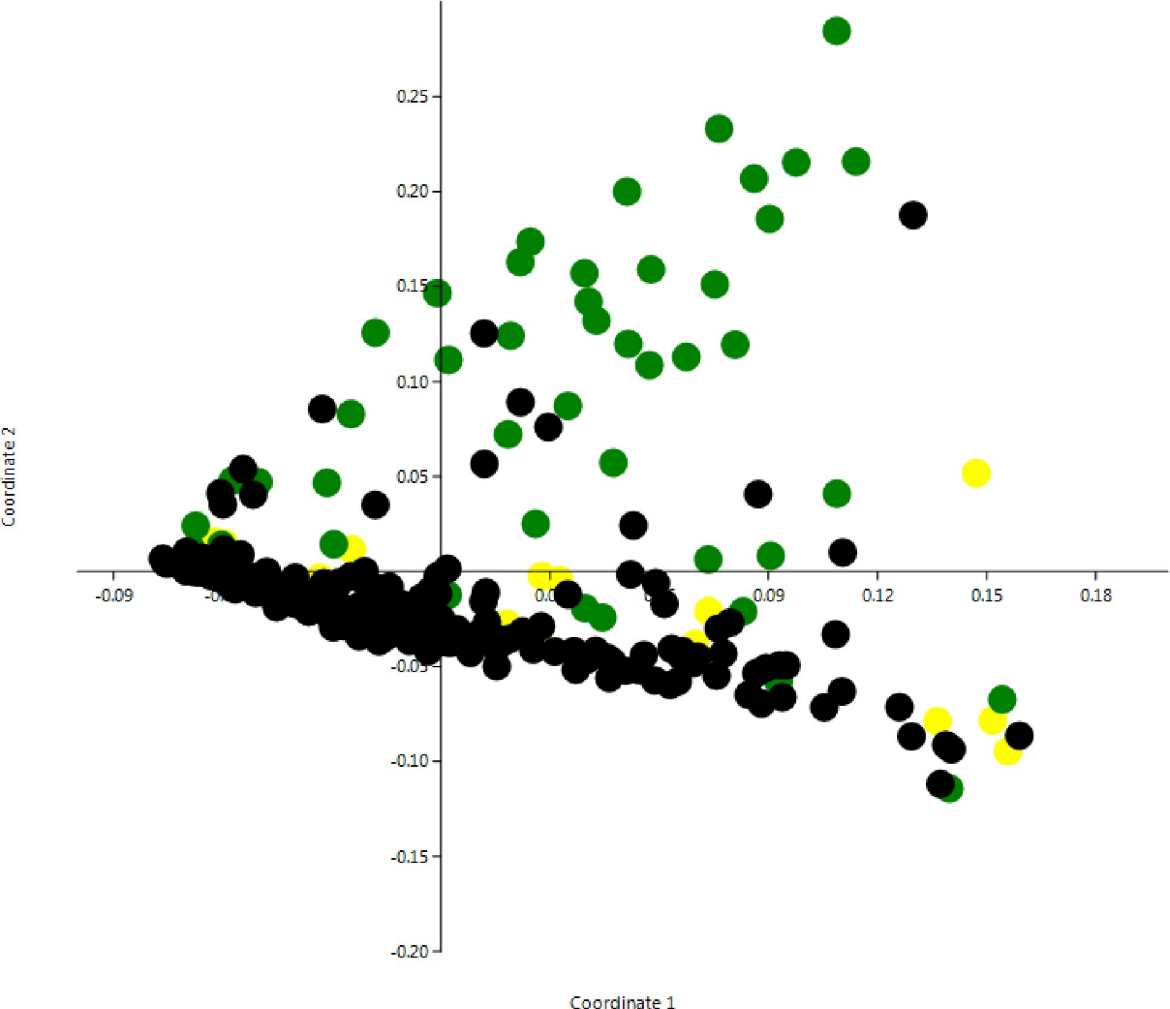
Principal coordinates analysis with Bray-Curtis similarity index of milk samples in the study (n = 278). Samples are color-coded based on bacterial growth; black = no growth, green = growth of *Corynebacterium* spp, yellow = growth of mixed bacterial flora.

A principal coordinate analysis (PCoA) based on Bray Curtis distances was applied on the sequence and culture dependent data to search for clustering patterns among the samples. The PCoA revealed that growth of *Corynebacterium* spp. was a major factor affecting dissimilarity in the milk samples (Fig 2). An analysis of similarity (ANOSIM) revealed that samples with bacterial growth (*Corynebacterium* or mixed flora) were significantly different from other samples (S3 Table).

**Fig 3:**
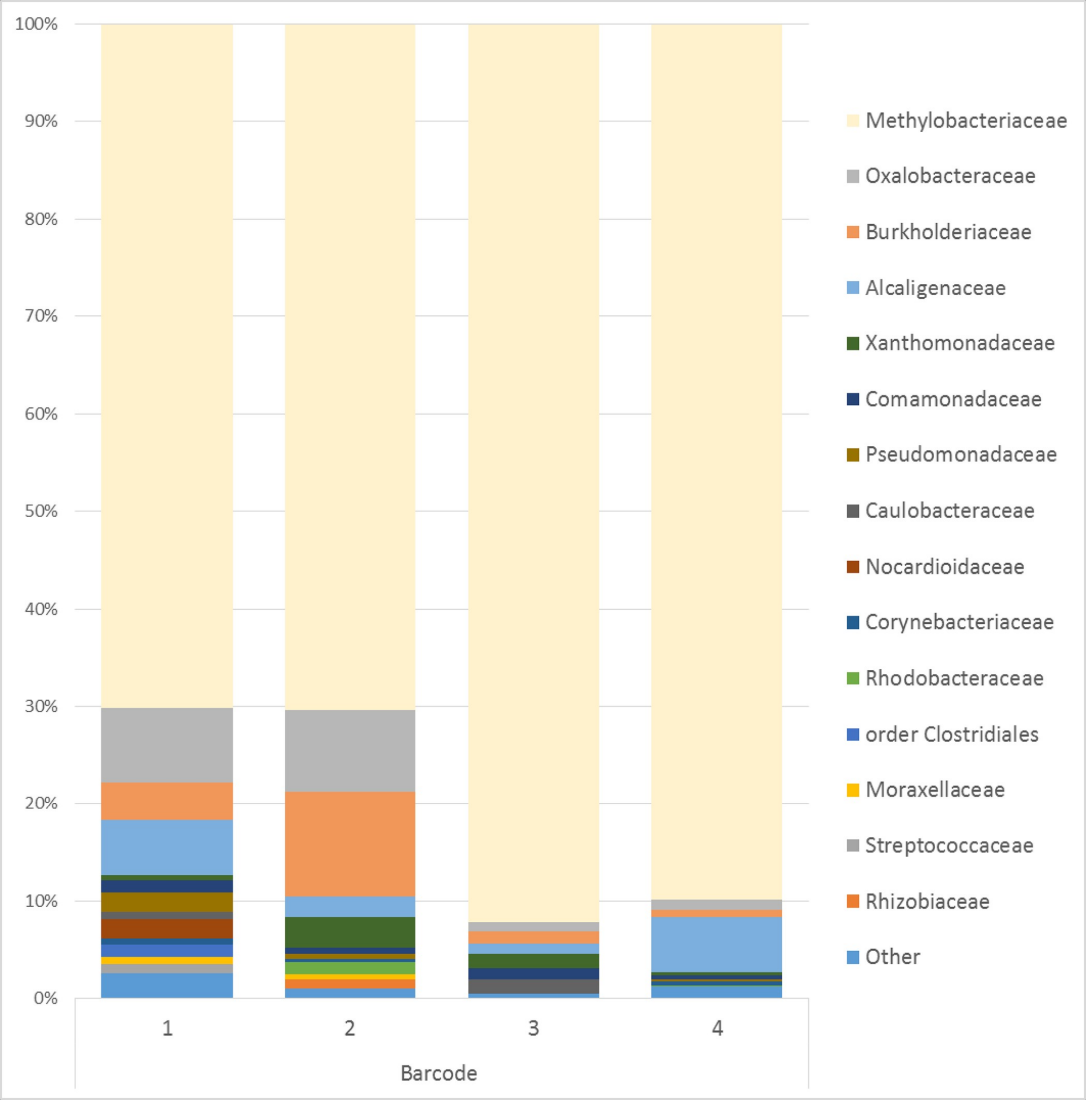
Relative abundance of the 15 most common families or order of bacteria found in NTC. 14 no-template DNA extraction controls (NTC’s) were individually processed and barcoded with a limited number of barcodes, 7 NTC’s marked with 4 different barcodes were included in sequencing.

After DNA isolation and PCR amplification, seven out of 14 NTC had measurable amounts of DNA and were subsequently sequenced. Within the four barcodes used for the sequenced NTC, 47 different taxa were identified. The most predominant genus in the NTC’s, *Methylobacterium*, was present in all NTC’s and represented 70.0–92.2% of the data (Fig 3). In addition, *Achromobacter*, *Burkholderia*, *Corynebacterium*, *Pseudomonas*, *Stenotrophomonas* and unclassified genus in the family Oxalobacteraceae and Comamonadaceae were present in all sequenced NTC.

Since *Methylobacterium* was detected in all milk samples and in all NTC’s we investigated if there were differences in (*Methylobacterium)* abundance, between DNA extraction batches with and without detectable amounts of DNA in the NTC. Regardless if the NTC for a specific DNA extraction batch contained or did not contain DNA, *Methylobacterium* was the most predominant genera in the associated milk samples. Moreover, there was no difference in relative abundance of *Methylobacterium* between milk samples prepared in DNA extraction batches with or without measurable amounts of DNA in NTC (p-value 0.75, t-test).

**Fig 4:**
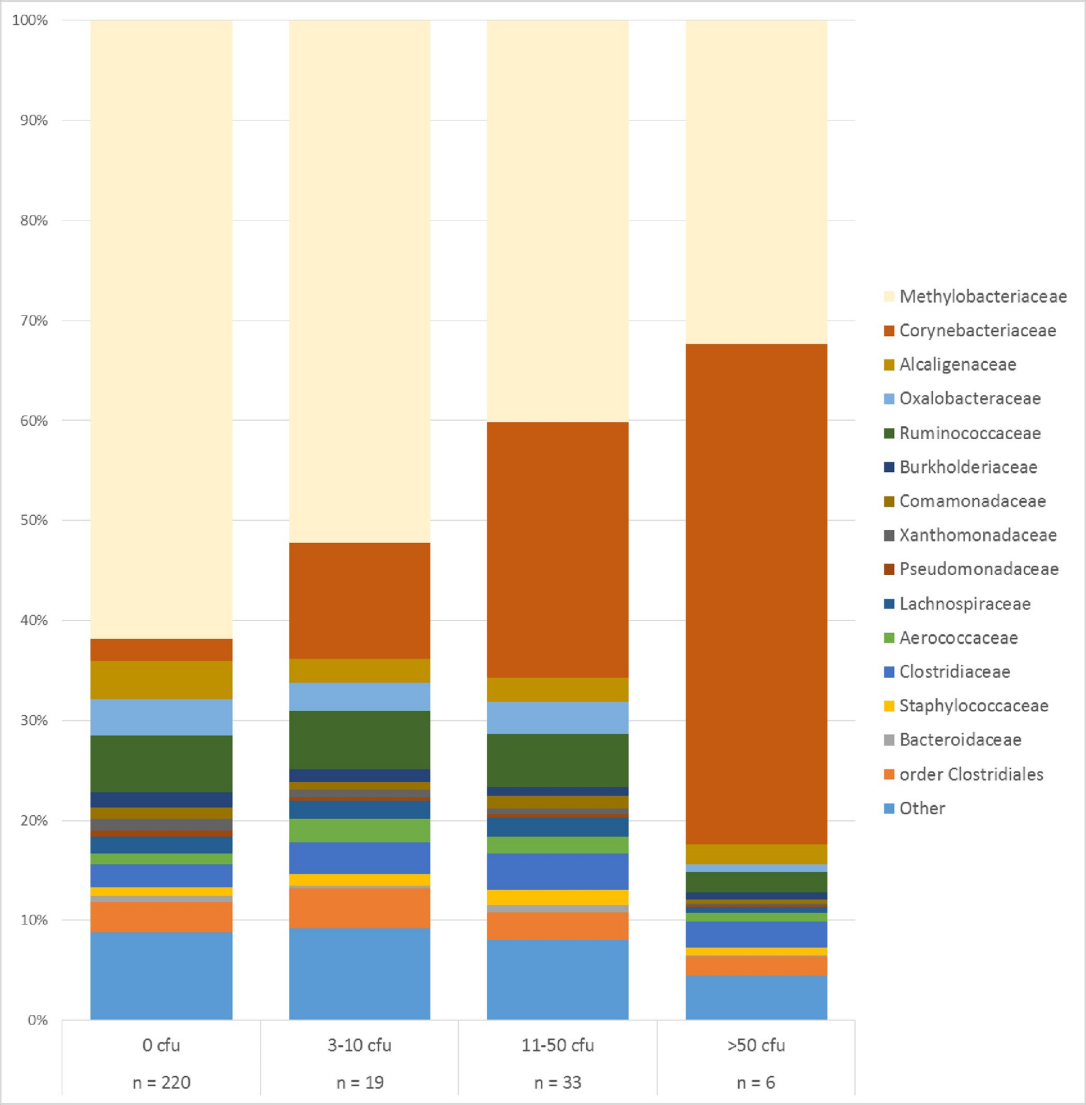
Relative abundance of the 15 most common families or order of bacteria found in bovine milk samples. Milk samples (n = 278) are grouped by number of colony forming units (CFU) in 10μl milk. Presence of *Corynebacterium* spp. was confirmed by culture and found in 44 milk samples.

There was an association between the proportion of *Methylobacterium* and number of bacteria determined by culturing. In milk samples with no bacterial growth after 48 hours, *Methylobacterium* was the most predominant taxa with an average abundance of 61.8%. In samples with abundant growth (i.e. >50 CFU/10μl) *Methylobacterium* was present in significantly lower proportions (P<0.01, t-test) with an average abundance of 32.3% (Fig 4), these samples were instead dominated by *Corynebacterium*, which is in agreement with what was found on the agar plates. There was a decrease in number of identified taxa per milk sample with increased bacterial growth/biomass, a total of 460 different taxa were identified in milk samples with no bacterial growth while a total of 94 different taxa were identified in milk samples with abundant bacterial growth (>50 CFU/10μl milk).

### Mock community as positive control

**Fig 5:**
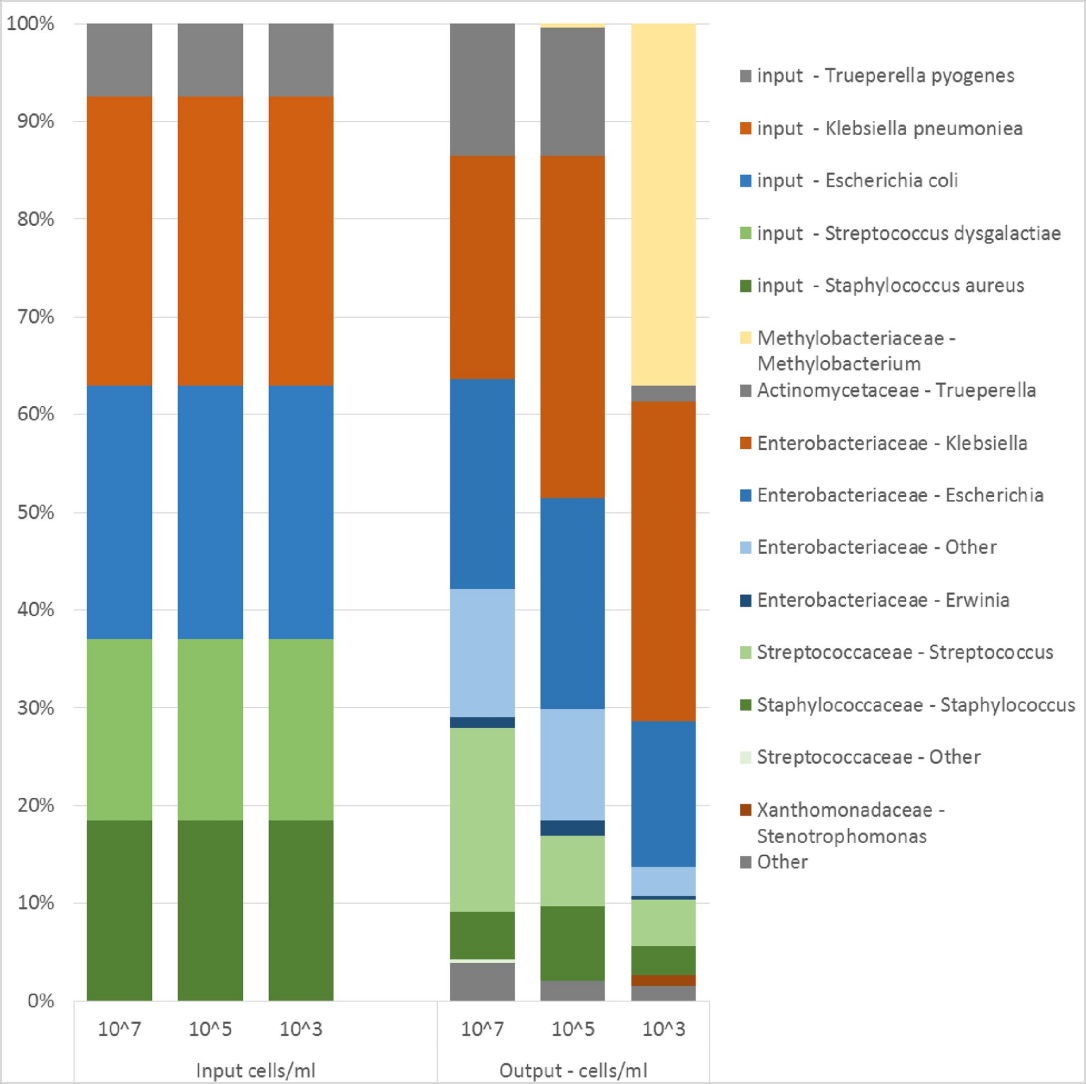
Relative abundance of input cells and the 10 most common genera found by sequencing in a mock community. Input bacterial cells, corrected for number of 16S rRNA genes per species (left), and sequencing results (right) from a bacterial mock community at three different dilutions of a bacterial mix created from five commonly occurring udder pathogens.

The microbial analysis of the three mock community dilutions revealed the presence of a total of 21 different taxa. More than 96% of the sequence data in the two highest concentrations (5x10^7^ and 5x10^5^ cells/mL) were associated with the input bacteria whereas in the lowest concentration (5x10^3^ cells/mL) only 60% of the sequence data originated from input bacteria. In the lowest concentration (5x10^3^ cells/mL) *Methylobacterium* represented 37% of the total abundance (Fig 5), while this species accounted for less than 0.5% of the sequences when the bacterial concentration was higher than 10^5^ cells/mL. There were also indications that sample processing (DNA extraction, PCR, choice of primer etc.) influenced the proportions of the taxa within the mock community. Gram-positive *Staphylococcus aureus* and *Streptococcus dysgalactiae* became less abundant in the sequence data compared to input, while *Trueperella pyogenes* became more abundant than expected. The Gram-negative *Escherichia coli* and *Klebsiella pneumoniae* became more abundant in the sequence data than input and were correctly classified at the family level (*Enterobacteriaceae*). However at genus level *Escherichia coli* and *Klebsiella pneumoniae* where classified as *Klebsiella*, *Erwinia*, *Escherichia* and “other”.

### Effect of data filtration and identified contaminants

An analysis of sequence data identified genera that represented >1% of the total relative abundance in a NTC as contaminants. Nine taxonomic families were found to contain at least one contaminant and were excluded from the data set. Consequently, the filtered data set did not contain any genera from the families; *Alcaligenaceae*, *Burkholderiaceae*, *Caulobacteraceae*, *Methylobacteriaceae*, *Nocardioidaceae*, *Oxalobacteraceae*, *Pseudomonadaceae*, *Rhodobacteraceae* or *Xanthomonadaceae*, a total of 39 genera in the data set belonged to these families. Contamination filtration led to a 72% reduction in available data from milk samples leaving 622 839 sequence reads for further analysis. The number of identified taxa decreased from 487 in the original data set to 438 in the filtered data set. After data filtration *Corynebacterium* and unclassified genus in the *Ruminococcaceae* and *Clostridiaceae* family were the most abundant genera, together they represented 33% of total abundance and were present in 94%, 96% and 72% of the milk samples respectively.

In order to study how the filtration method influenced the relation between samples, UniFrac distances between samples collected consecutively from the same cow and quarter were compared with UniFrac distances between randomly selected samples in the data set. Before filtration there was no difference in the UniFrac distance between consecutive and random comparisons (p-value 0.30, t-test). However in the filtered data set there was a significant difference between consecutive and random comparisons with larger UniFrac distances for random comparisons (p-value <0.01, t-test).

The recently published “decontam” R-package [30] was also used to identify contamination. The strict (0.5) threshold settings in the prevalence-based contaminant identification in the “decontam” R-package identified 32 contaminating taxa. Ten taxa were identified as contamination by both protocols, a list of identified contaminants is available in S2 Table, *Corynebacterium* was but *Methylobacterium* was not identified as a contaminant by the “decontam” R-package.

## Discussion

The animal experiment for this study was designed to compare the milk microbiota within quarter over time, as well as between quarters and between animals over time. A large proportion of quarters had a similar bacteriological finding by culture throughout the study period indicating that bacteria findings by culture dependent methods were stable over time. *Corynebacterium* spp. was the most commonly isolated bacteria by culture and was repeatedly detected in milk from the same quarters adding further support that bacteriological response was stable over time. We aimed to study the composition and temporal stability of the milk microbiota using 16S amplicon sequencing. Despite a very careful treatment of the samples, with all DNA isolations carried out in a laminar air flow hood pretreated with UV light and cleaned with both 10% bleach and 70% ethanol, contamination from reagents and the laboratory environment had a pronounced effect on the results. Due to this contamination, characterization and assessment of temporal stability of the bovine milk microbiota via 16S rRNA sequencing proved difficult and is further discussed below.

When bacterial cell count determined by culture dependent analysis or manual counting was below 10^4^ cells per mL, contaminating taxa became more dominant. This was observed both in sequence data generated from a created mock community as well as in milk samples where concentration of bacteria was determined by a culture-based approach. When the bacterial concentration in a created mock community corresponded to 5x10^3^ cells/mL *Methylobacterium*, the major contaminant in this study, represented 37% of the sequences. Similar proportions of *Methylobacterium* was identified in milk samples when the bacterial concentration corresponded to 10^3^−10^4^ cells/mL. Noteworthy is an observed correlation between the relative abundance of *Methylobacterium* and abundance of bacterial growth in milk samples, where *Methylobacterium* became more abundant with fewer viable bacteria (Fig 4). These results are in line with previously published data from Salter *et al*. and Glassing *et al*. [6, 7] who reported thresholds of 10^3^–10^4^ bacterial cells/mL and 10 *E*. *coli* equivalent genomes per μl respectively. Thus, the evidence that low bacterial biomass 16S-based microbiota studies are prone to be contaminated is increasing.

Randomly occurring DNA contamination in a laboratory environment is challenging to overcome and milk samples can easily become contaminated either at sampling in the barn, or during laboratory sample processing. In this experiment precaution was taken to minimize and characterize contamination arriving from different steps but measurable amounts of DNA occurred in every other NTC from DNA extraction. Due to the small bacteriological biomass in the milk samples it was necessary to use many PCR-cycles. Using many PCR-cycles may enhance the impact of a contamination and introduce PCR-artefacts such as chimera sequences but was inevitable in this study. However, there was no statistical difference in relative abundance of *Methylobacterium* in milk samples prepared in batches where measurable amounts of DNA could be found in the NTC, compared with batches where no DNA could be found in the NTC. This led us to the conclusion that absence of visible bands or measurable amounts of DNA in NTC´s does not necessarily imply absence of contamination.

*Methylobacterium* is a genus consisting of 52 species of aerobic Gram-negative bacteria that are slow growing, commonly isolated from soil, leaf surface and fresh water and have capacity to form biofilms [33]. *Methylobacterium* has been reported to cause colonization and infection in immunocompromised humans [34] and has previously been reported as a contaminant in microbiome studies [6, 35]; to our knowledge *Methylobacterium* has never been isolated from milk of dairy cows.

Different DNA extraction methods can affect and skew the relative abundance of bacteria present in a mock community and some DNA extraction methods are more prone to introduce contamination [18, 36]. Further, in each step to prepare samples for sequencing there is a risk that biases are introduced (thoroughly discussed by Pollock *et al*. [17]). We used the Power food DNA extraction kit from MO BIO since this according to the literature [37] and personal experience yielded most DNA from the milk samples. From the sequenced mock community we noticed that the method introduced some biases in the distribution of taxa. Sequencing of the mock community led to an overestimation of Gram-negative bacteria and an underestimation of two out of three Gram-positive bacteria (Fig 5) an effect that might be related to the DNA extraction method used. We also noticed that partial sequencing of the 16S rRNA gene might not be sufficient for correct annotation of all present bacteria since *E*. *coli* and *K*. *pneumoniae* were annotated into four different genera, although, all within the *Enterobacteriaceae* family.

Identification of contaminants in this study was based on presence of a taxa in NTC and a threshold of >1% relative abundance. Davis *et al*. [30] reason in a similar manner for the prevalence-based contaminant identification in the R-package “decontam”. Accordingly; prevalence of contaminants will be higher in negative controls than in true samples due to the absence of competing DNA in the sequencing process. The prevalence-based contaminant identification in the “decontam” R-package also include a stricter threshold option that will identify all sequences that are more prevalent in negative controls than in positive samples as contaminants. Here, *Methylobacterium* was not identified as a contaminant by the “decontam” R-package, likely due to the presence of *Methylobacterium* in all samples and high prevalence in the milk samples. In conclusion, the “decontam” R-package is a highly useful tool and complement for identification of contaminating taxa.

Evaluation of the data filtering of contaminants in this data set was made under the assumption that a milk microbiota stable over a short time period exists, in the absence of disease and major environmental changes. This assumption was based on the fact that in the bovine udder each quarter (mammary gland) functions as a separate unit. Intra-mammary infection often occurs in a quarter with no immunological or bacterial response in neighboring quarters. Studies of the bovine milk microbiota have confirmed that the microbiota in two quarters within the same cow can be substantially different [2, 11] and also that the microbiota profile of quarters within the same cow are more similar to each other than quarters of other cows [38]. It has also been shown that the human milk microbiota often, yet not always, is stable over time [39]. Thus, we expected the difference in microbiota between two samples to be smallest if they were taken from the same quarter from two consecutive sampling points that had the same bacteriological response by culture. With the weighted UniFrac dissimilarity matrix created in QIIME for the original and the filtered data, distances for consecutive samplings in bacteriologically stable quarters were compared to distances for randomly selected comparisons in the data set. In the original data set there was no difference between consecutive and random comparisons, while in the filtered data set there was a significant difference between consecutive and random comparisons, with larger similarity between consecutive comparisons. This demonstrates that even if the data filtration contribute to a substantial reduction in sequence data it can improve the possibility to find biologically relevant associations in milk microbiota data.

In this study *Corynebacterium* was the most commonly isolated bacteria in milk samples by culture but *Corynebacterium* DNA was also present in all NTC. With the threshold set to >1% abundance in a NTC for a genera to be categorized as a contaminant, *Corynebacterium* did not meet the requirements and was subsequently not filtered from the data set, consequently *Corynebacterium* became the most abundant genus in the data after filtration. Interestingly *Corynebacterium* isolated by culture was a major factor affecting dissimilarity before filtration even though it was not very abundant (S3 Table). Of the nine taxonomic families that were filtered from the data set, none is known to contain major mastitis causing pathogens. In the family *Pseudomonadaceae*, one genus (*Pseudomonas aeruginosa*) is known to cause mastitis in dairy cows but is considered a rare udder pathogen in Sweden [40]. A similar strategy to exclude contaminating taxa used here was discussed by Glassing *et al*. [7] but rejected due to too large data loss and loss of known endogenous taxa. The method for filtration used herein did indeed lead to a great reduction in available data but had a small effect on the number of identified taxa and only had a minor impact on known endogenous taxa in the bovine milk microbiota.

In this study we have shown that the impact of contamination in samples with a low biomass can conceal biologically relevant associations. Further we conclude that proper identification of the contaminants is necessary in order to evaluate the overall impact of the contamination, and that absence of measurable amounts of DNA in negative controls does not imply absence of contamination.

## Supporting information

S1 Fig Box plot of the 10 most abundant genera separated by bacterial growth in 10 μl of milk. Relative abundance of the ten most abundant genera before data filtration separated by bacterial growth in 10 μl of milk. The 25–75 percent quartiles and median value are shown within the box, whiskers represent value less than 1.5 times box height, values 1.5–3 times box height are shown as circles and values >3 times box height are shown as stars.
(PNG)

S1 Table Sample information. (XLSX)

S2 Table List of contaminants identified as >1% prevalence in NTC or by the “decontam” R-package. (XLSX)

S3 Table ANOSIM between samples classified by type of bacterial growth. One-way ANOSIM with Bonferroni corrected p-values based on BrayCurtis similarity index, samples are classified based on the type of bacterial growth identified by culture. One sample with growth of *Staphylococcus* omitted.
(DOCX)
