# Supplementary figures and images for: Microbiota data from low biomass milk samples is markedly affected by laboratory and reagent contamination
Source: PLoS One. 2019 Jun 13;14(6):e0218257. doi: 10.1371/journal.pone.0218257 (PMC6564671; doi:10.1371/journal.pone.0218257)

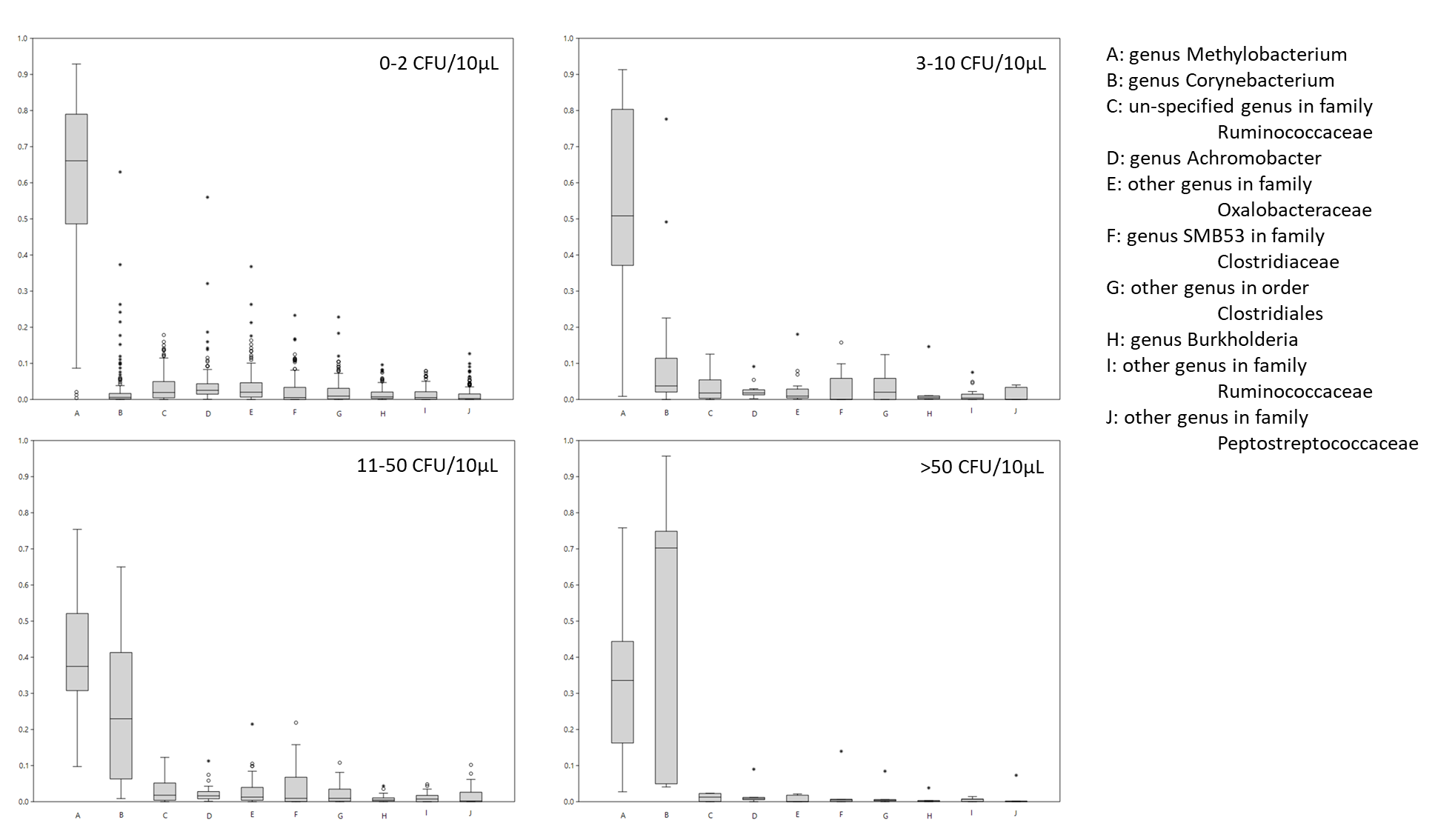

Supplement: S1 Fig — Relative abundance of the ten most abundant genera before data filtration separated by bacterial growth in 10 μl of milk. The 25–75 percent quartiles and median value are shown within the box, whiskers represent value less than 1.5 times box height, values 1.5–3 times box height are shown as circles and values >3 times box height are shown as stars. (PNG) [file pone.0218257.s001.png]
