# Supplementary material for: Microbiota data from low biomass milk samples is markedly affected by laboratory and reagent contamination
Source: PLoS One. 2019 Jun 13;14(6):e0218257. doi: 10.1371/journal.pone.0218257 (PMC6564671; doi:10.1371/journal.pone.0218257)
Supplement: S3 Table — One-way ANOSIM with Bonferroni corrected p-values based on BrayCurtis similarity index, samples are classified based on the type of bacterial growth identified by culture. One sample with growth of Staphylococcus omitted. (DOCX) [file pone.0218257.s004.docx]

**S3 Table. ANOSIM between samples classified by type of bacterial growth.** One-way ANOSIM with Bonferroni corrected p-values based on BrayCurtis similarity index, samples are classified based on the type of bacterial growth identified by culture. One sample with growth of *Staphylococcus* omitted.

|  | No growth | Corynebacterium | Mixed flora |
| --- | --- | --- | --- |
| No growth |  | 0.0003 | 0.0483 |
| Corynebacterium | 0.0003 |  | 0.0171 |
| Mixed flora | 0.0483 | 0.0171 |  |
